# Topical 100% Serum Eye Drops for Treating Corneal Epithelial Defect after Ocular Surgery

**DOI:** 10.1155/2013/521315

**Published:** 2013-07-30

**Authors:** Kaevalin Lekhanont, Passara Jongkhajornpong, Lulin Choubtum, Varintorn Chuckpaiwong

**Affiliations:** ^1^Department of Ophthalmology, Ramathibodi Hospital, Mahidol University, Rama VI Road, Rajathevi, Bangkok 10400, Thailand; ^2^Ramathibodi Research Center, Ramathibodi Hospital, Mahidol University, Rama VI Road, Rajathevi, Bangkok 10400, Thailand

## Abstract

The purpose of this study was to investigate the efficacy and safety of topical 100% serum eye drops for corneal epithelial defect after ocular surgery. A total of 181 patients who received topical 100% serum therapy for the treatment of corneal epithelial defect following several different types of ocular surgery were recruited into this study. Each patient already failed conventional medical therapy before being prescribed 100% serum eye drops. Slit-lamp biomicroscopic examination with fluorescein staining was performed at baseline and all follow-up visits. The main outcome measures were the rate of complete healing of the corneal epithelial defect and incidence of adverse events. One hundred and seventy-eight eyes (98.34%) received autologous serum eye drops, and 3 (1.66%) received allogeneic serum eye drops. The overall success rate of treating persistent postoperative epithelial defect using 100% serum eye drops was 93.92% (95% CI 0.88–0.98). The median time to complete corneal epithelialization was 4 days (95% CI 4-5). Adverse reactions were observed in 3 patients (1.66%), including sticky sensation with minimal eye discomfort and asymptomatic trace corneal subepithelial infiltration. No serious complications were reported. In conclusion, 100% serum eye drops are effective, safe, and tolerable for treating postoperative corneal epithelial defect following ocular surgeries.

## 1. Introduction

Autologous serum application for the treatment of ocular surface disease has been dated back at least to 1975 when it was used via a mobile perfusion pump to treat ocular alkali injuries [1]. Later, in 1984, Fox et al. first described a successful use of autologous serum as an eye drop in patients with dry eye [2]. However, it was not until the late 1990s that scientific interest in the use of autologous serum eye drops emerged due to the research of Tsubota et al. [3, 4]. Initial works were subsequently continued by many pioneers over the following years. Today, this treatment modality has become popular and gained widespread acceptance as an adjuvant therapy for various ocular surface disorders, including dry eye, persistent epithelial defect, neurotrophic keratopathy, recurrent erosion syndrome, and superior limbic keratoconjunctivitis. Topical autologous serum has also been found to be beneficial in promoting graft reepithelialization following penetrating keratoplasty [5–7]. Additionally, serum eye drops appeared to allow corneal epithelial wounds after vitreoretinal surgery to heal effectively and faster than artificial tears [8, 9]. 

The concept of using serum as natural tear substitutes is based on the finding that serum contains essential ocular surface nutrients, such as epidermal growth factor (EGF), transforming growth factor- (TGF-)*β*, platelet-derived growth factors (PDGF), neurotrophic factors, fibronectin, vitamin A, vitamin E, cytokines, and bacteriostatic components, which are not present in artificial tears but exist in normal tears. The pH, osmolality, and biomechanical characteristics of serum also resemble those of natural tears. In addition, serum is generally free of preservatives, stabilizers, or additives [10]. However, the concentrations of the epitheliotropic factors in serum and tear fluids are dissimilar. Since TGF-*β* is known to have antiproliferative properties in a dose-dependent manner and its level in serum is approximately 5 times higher than that in tears, autologous serum eye drops are usually prepared as a 20% dilution to prevent this potentially harmful effect [11–13]. Nonetheless, dilution may reduce the concentration of other beneficial factors at the same time, particularly EGF and fibronectin, which are proven to support proliferation and migration of corneal epithelial cells [14]. Although most published studies have reported the use of 20% autologous serum eye drops for treating a number of ocular surface conditions, some demonstrated good results in terms of both efficacy and safety at higher concentrations of 50–100% as well [6, 15–17]. The undiluted autologous serum has previously been shown to be more effective than 20% serum in the epithelial healing process of mechanical corneal ulcers in animal study [18]. Additionally, undiluted serum resulted in better human epithelial cell migration than diluted one, probably because of the higher concentration of fibronectin [19]. Therefore, we conducted a prospective study to investigate the therapeutic effects of topical 100% serum eye drops for corneal epithelial defect after ocular surgery. 

## 2. Methods

### 2.1. Study Design

This was a single-center, prospective, and interventional study, assessing the ability of topical 100% serum eye drops in the management of corneal epithelial defect following various ocular surgeries. 

### 2.2. Participants

This trial was conducted between March 2008 and December 2012, at Ramathibodi Hospital, Bangkok, Thailand. The study protocol adhered to the tenets of the *Declaration of Helsinki* and was approved by the ethics committee of Mahidol University School of Medicine. A total of 181 consecutive patients who received topical 100% serum therapy for the treatment of corneal epithelial defect after several different types of ocular surgery were recruited into this study. The eligibility criteria included

1. having uneventful ocular surgery;
2. having total corneal epithelial defect on 1 day following surgery;
3. persistent postoperative epithelial defect longer than 1 week;
4. receiving standard postoperative drug regimen tailored to the individual, taking into account the type of surgery and indication for surgery;
5. no history of allergy to the medications used in this study;
6. good compliance with the study regimen and availability for the duration of the entire study period;
7. nonpregnant or lactating women.

Each patient already failed conventional medical treatments, for example, preservative-free artificial tears, lubricating gel/ointment, anti-inflammatory agents, punctal plugs, and bandage contact lenses, before being prescribed 100% serum eye drops. All patients were informed regarding the advantages, disadvantages, and potential side effects of this modality such as sticky sensation or ocular discomfort and the possible risks of the procedure. Written informed consent was obtained prior to enrollment. Except for nonpreserved artificial tears (0.18% sodium hyaluronate) which were replaced with serum eye drops, concurrent therapy consisted of needed preexisting medications in similar or decreased dosages, including topical corticosteroids, antibiotics and/or antifungals, or antiglaucoma drugs.

Persistent or delayed healing epithelial defect has been defined as an epithelial defect that does not heal within the expected time course [20]. It has been suggested that the length of time required for healing directly correlated with the length of time a persistent epithelial defect has been present [19]. Some studies applied the serum earlier in the disease course rather than as a last resort since the patients might derive as much benefits as those who were recalcitrant to standard treatments [9, 21]. Furthermore, corneal graft re-epithelialization after corneal transplantation usually takes no longer than 1 week [22]. Therefore, in this study, the serum eye drops were used if the postoperative corneal epithelial defect persisted longer than 1 week to minimize the risks of infection, subepithelial haze, corneal melting, and perforation which could occur with prolonged duration of epithelial defects. 

### 2.3. Interventions

Autologous serum eye drops were prepared according to the standardized protocol of Geerling et al. and Liu et al. [14, 19]. Briefly, confirmation of the absence of human immunodeficiency virus, syphilis, and hepatitis B and C infections in every patient must be carried out. Venipuncture was performed at the antecubital fossa under aseptic conditions, and 50–100 mL of whole blood was collected into sterile tubes without clot activator or anticoagulant. The tubes were left standing for 2 hours at room temperature (18–25°C) in an upright position for complete clotting, followed by centrifugation at 3000 g for 15 minutes. The supernatant serum was transferred aseptically into sterile plastic tubes in a laminar air flow hood. The volume retrieved was determined and filter sterilized (0.2 *μ*m) with no dilution. Portions of 1-2 mL were aliquoted into individual colored eyedropper bottles. The bottles were sealed and labeled with the name, hospital number of the patient, the date of production, and identification as serum for topical use in the eye and dosage frequency. All preparations were performed by only one well-trained medical staff, in a semisterile environment, using single-use sterile supplies and a biologic safety cabinet, optimally equipped with ultraviolet light protection to avoid microbial contamination and vitamin A degradation. No more than one blood sample from one person was manipulated at the same time. Following this protocol, 50 mL of whole blood yielded approximately 20–25 mL of serum. Allogeneic serum donated from a family member was used when a patient's own serum was unsuitable or unavailable for processing into autologous serum eye drops, including patients with positive viral or syphilis serology, septicemia, significant cardiovascular diseases, and severe anemia and infants or the extremely elderly.

Patients were instructed to store all bottles of serum in their freezer ideally at −20°C and thaw one bottle for use at a time in the refrigerator at +4°C. A shelf life for the thawed bottle was set at 24 hours; it was kept in the refrigerator (+4°C) after each use and discarded at the end of the day. The serum eye drops must be used within 3 months after date of production. Even though some growth factor peptides such as EGF, TGF-*β*, and insulin-like growth factor 1 (IGF_1_) were relatively temperature and time resistant, substance P (SubP) and calcitonin gene-related peptide (CGRP) significantly degraded at −15°C in 6 weeks, +4°C in 24 hours, and +25°C in 6 hours [23]. However, endotoxin levels did not significantly increase at +25°C in 24 hours [23]. Consequently, if the domestic freezer and refrigerator had no thermometer or the temperatures seemed to be greater than the optimum, patients were asked to place the bottles inside and finish the eye drops within a shorter storage time which was 12 hours rather than 24 hours for an active bottle and 1.5 months instead of 3 months for frozen bottles to ensure stability and sterility of the serum tear. The frequency of instillation was every 2 hours while awake until the defect was healed. After epithelial healing achieved, the serum eye drops were gradually tapered to 4 times daily for an additional week or until the serum ran out.

During administration of serum eye drops, the patients were evaluated every day for admitted patients and every 1-2 days for outpatients until there was a total corneal epithelialization. Slit-lamp biomicroscopic examination with fluorescein staining was performed at baseline and all follow-up visits. The size of the epithelial defect was measured in two linear dimensions, the longest linear diameter and the largest one perpendicular to it, within the confines of the epithelial defect with the help of fluorescein staining and a standard slit lamp under 10x magnification [24].

### 2.4. Outcome Measures

Primary outcome measures were complete healing of the corneal epithelial defect and incidence of adverse events. Secondary outcomes were time to complete corneal epithelialization, probability of healing at certain time points during followup, and the relationship between subject characteristics and outcomes. Success was defined as the complete closure of corneal epithelial lesions. Treatment failure was defined if (a) there was no objective improvement in corneal epithelial healing within 1 month of serum therapy, (b) the lesion was enlarging or worsening, or (c) surgical intervention was necessary. 

### 2.5. Statistical Analyses

Statistical analyses were performed with the statistical software package STATA version 11.1 (Stata Corp, College Station, TX, USA). Mean and standard deviation (SD) or median and range were used to describe continuous data. Frequency and percentage were used for categorical data. For prevalence estimates, the 95% confidence intervals (CIs) are provided. Kaplan-Meier survival analysis was used to estimate the success rate and overall probability of the event (complete corneal epithelialization) occurring at different time points. Related factors for corneal reepithelialization were first examined in univariate analyses by the log-rank test and simultaneously in a Cox proportional hazards regression analysis. Variables with *P* < 0.05 in univariate analysis were selected for Cox regression multivariate analysis. A *P* < 0.05 was considered to be statistically significant.

## 3. Results 

Of the 181 eyes, 178 eyes (98.34%) received autologous serum eye drops, and 3 (1.66%) received allogeneic serum eye drops. The reasons for using allogeneic serum eye drops in some cases included human immunodeficiency virus (HIV) infection (1) and elderly with multiple systemic diseases (2). Allogeneic serum was taken from the patient's spouse (1) and offspring (2). The donors were tested for blood-borne diseases such as HIV, hepatitis B and C viruses and syphilis using standard blood bank screening tests to ensure that their serum was suitable for processing into serum eye drops. All patients were followed up for a minimum of 3 months. Patient baseline characteristics are summarized in Table 1. The majority of patients underwent corneal transplantation surgery either penetrating or lamellar keratoplasty (151 eyes, 83.43%), followed by vitreoretinal surgery (16 eyes, 8.84%), and laser corneal surgery (6 eyes, 3.31%), respectively. A history of DM was found in 45 patients (24.86%). Most patients had blood draws only once (160 patient, 88.40%). The remaining required multiple blood draws secondary to delayed response or treatment failure. 

**Figure 1:**
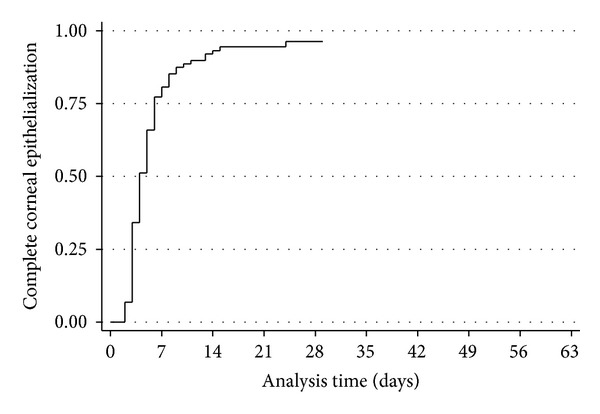
Kaplan-Meier survival analysis shows the time to complete corneal epithelialization in patients receiving autologous/allogeneic serum eye drops.

The overall success rate (complete corneal epithelialization) of treating persistent postoperative epithelial defect using 100% serum eye drops was 93.92% (170/181 eyes; 95% CI 0.88–0.98). The median time to complete corneal epithelialization was 4 days (95% CI 4-5) (Figure 1). The probability of achieving complete corneal epithelial healing during the followup is shown in Table 2. The presence of DM and type of operation did not have significant impacts on corneal re-epithelialization time (*P* = 0.34 and 0.21, resp.). Three patients undergoing PK for herpetic neurotrophic corneal scar (1), graft failure after resolution of acanthamoeba keratitis (1), and multiple graft failure with secondary Sjögren's syndrome caused by rheumatoid arthritis (1) developed new corneal epithelial defect after initially complete epithelialization and weaning of serum eye drops. The serum eye drops were reintroduced, and the condition improved significantly 1 week later. The summary of the clinical details of 11 patients (6.08%) who did not respond to serum eye drops is presented in Table 3. These patients had signs of worsening including enlarged epithelial defect and stromal thinning 2 weeks after treatment. The dosage of serum tear was increased to every hour, but the lesion was not ameliorated and finally required surgical intervention. 

Adverse reactions were observed in 3 patients (1.66%) receiving autologous serum eye drops. Two patients had sticky sensation with minimal eye discomfort, and 1 patient developed asymptomatic trace corneal subepithelial infiltration, but none of these patients discontinued treatment. The infiltrates disappeared after the epithelium healed, leaving no haze or scar. No side effects were seen in patients receiving allogeneic serum eye drops. Also, no serious complications such as infectious keratitis were reported during the entire study period. 

## 4. Discussion 

Over the years, there has been reliable evidence in the literature regarding the success in the treatment of ocular surface disease with the use of serum eye drops [25–36]. However, most of these studies are retrospective and run different protocols for the production of the serum. The concentration of serum eye drops in these studies also varied (from 20% to 100%). A variety of steps of serum production, including clotting time, centrifugation time and force, dilution, and diluents, might yield various concentrations of the epitheliotropic factors in the resulting serum, and these differing levels can have different effects on the ocular surface healing process [37]. The most preferred concentration applied in previous studies is 20%. The rationale for diluting the serum 1 : 5 is used to decrease the concentration of TGF-*β* in serum to a level equivalent to that in natural tears because the very first study, using low centrifugation speed, revealed a fivefold greater concentration of TGF-*β* in serum than in tears, possibly retarding epithelial wound healing [3]. Nonetheless, a high centrifugation force was used for serum preparation in the later study, and they found a much lower concentration of TGF-*β* than was seen in the early report [14]. In this study, undiluted serum eye drops were used instead of diluted ones since we believed that 100% serum eye drops would provide higher concentration of growth factors as well as reduce the risk of dropper bottle contamination due to less manipulation of the serum. With the optimized manufacturing protocol [14], the serum and tear concentration of TGF-*β* are supposed to be similar, and dilution may not be necessitated.

Our study demonstrated overall high success rates with 100% serum eye drops in the management of corneal epithelial defect following various ocular surgeries. These favorable results from this large-scale study resemble those found in small pilot studies using higher concentration eye drops (50–100%) [6–8, 17]. Previous study evaluating the effect of routine use of postoperative 20% topical autologous serum in accelerating graft re-epithelialization revealed that 91.5% of patients had complete corneal epithelial healing within 2 weeks [5]. In this study, we started 100% serum eye drops when the corneal epithelial defect persisted for more than 1 week, not immediately following the surgery like previous study, and found that 93.2% achieved complete epithelial closure within 2 weeks after treatment. The similar high percentage of rapid healing even though the serum drops were applied later in this current study is possibly due to the use of higher serum concentration. Earlier therapy of greater serum concentration might be able to induce faster recovery. 

Despite the fact that most postoperative epithelial defects could finally heal in an acceptable time frame without the use of serum drops, shortening the healing time would help to minimize recovery time, decrease the risk of chronic epithelial defect-related complications, and reduce the length of stay and hospitalization costs, especially in patients with low socioeconomic status. 

From this observation, although we could not assert the superiority of undiluted serum over diluted serum eye drops, it implies that 100% serum clinically provides at least as effective as lower concentration drops in enhancing corneal epithelial wound closure and does not seem to have detrimental effects on the healing process. Another advantage of undiluted serum is the lower risk of contamination, particularly if the serum is prepared in unit dose for daily use. The addition of diluents or antibiotics to the compound to prevent microbial growth is unnecessary, thus reducing the costs of production of serum eye drops. Furthermore, the possible toxicity of diluents on corneal epithelial cells is totally avoided. 

However, the major drawbacks of using undiluted serum eye drops are the inconvenience of repeated blood draws, large volume of blood collection, and potential ocular irritation associated with extra viscosity of the eye drops. In this study, although high concentrations of serum proteins in 100% serum can alter the osmolarity and pH of the preparation [17], very few patients had ocular discomfort which was tolerable. Subclinical corneal infiltration was found in only 1 patient, probably caused by immune complex deposition similar to previous studies [6, 38]. Additionally, most patients healed quickly within 2 weeks, and 88.40% had a single venipuncture. Nonetheless, few patients took weeks to heal or developed new corneal epithelial defect after initially complete closure and stopping using serum eye drops. This means that some patients required serum eye drops for an extended period of time to prevent a recurrent epithelial breakdown after initial epithelialization. A continuation of diluted serum eye drops after withdrawal of undiluted serum might be a good choice to avoid any inconvenience and cost of repeated phlebotomy. Allogeneic serum is another viable option especially when a large quantity of blood is needed, and patients are medically unable to give blood samples. Nevertheless, it must be used extremely carefully because the serum includes not only beneficial factors for the ocular surface but also harmful and unknown infectious factors. Prior to starting allogeneic serum drops to our 3 patients, all methods of treatment for corneal re-epithelialization have been performed; however, the total corneal epithelial defect in graft still persisted 1 week after PK. One patient had HIV infection which was considered as a contraindication of autologous serum eye drops because of the risk of viral transmission to third parties [14]. The other 2 patients had multiple systemic diseases and believed that large amount venesection would result in further weakness. Hence, allogeneic serum eye drops from related donors had to be used as an alternative treatment. 

Unsurprisingly, 11 cases with preexisting definite diseases including total LSCD due to alkali injuries, ocular cicatricial pemphigoid, Stevens-Johnson syndrome, and postlimbus to limbus therapeutic keratoplasty for severe fungal infection were unresponsive to serum treatment. This approach improves the ocular surface environment but does not directly address the basic pathologic conditions of these diseases. Correcting and controlling the underlying illness is crucial. 

There are some limitations of this study. First, our study population predominantly consisted of patients with PK, and the sample size in other types of operations was relatively small. This may affect the statistical significance of the results. Second, no “washout period” was used, since we were trying to identify whether serum would have an additional effect on epithelial healing over conventional therapy, not comparing the efficacy of serum against standard treatments. Also, giving serum alone without continuing the other treatments may have caused harm to some patients. Third, the follow-up time was short, and information on disease recurrence was not gathered because most of our patients were referred from a long distance, and thus they were eventually returned to their local ophthalmologists for followup. 

In conclusion, the results of this study indicate that 100% serum eye drops are effective, safe, and tolerable for treating postoperative corneal epithelial defect following various ocular surgeries. Future research is needed to fully understand the short- and long-term effects of human serum on each stage of corneal healing and to determine the optimum concentration of serum for specific diseases. Prospective, randomized, masked, and controlled clinical trial to test the safety and efficacy of serum therapy at varying concentrations, with different preparations, and for various indications would guide the acquisition of the knowledge and strength to properly implement serum eye drops as a standard treatment modality.

## Figures and Tables

**Table 1:** Participant baseline characteristics.

Characteristics	Number (%)
Total patient numbers	181 (100)
Age (years)	
Mean ± SD	62.39 ± 14.28
Range	34–89
Sex	
Male	89 (49.17)
Female	92 (50.83)
Laterality	
Right eye	85 (46.96)
Left eye	96 (53.04)
Operations	
(i) Penetrating keratoplasty	99 (25.78)
(ii) Anterior lamellar keratoplasty	40 (10.42)
(iii) Pars plana vitrectomy with corneal epithelial debridement	16 (4.17)
(iv) Combined PK and cataract surgery	12 (3.13)
(v) Phototherapeutic keratectomy	6 (1.56)
(vi) Trabeculectomy with mitomycin C	3 (0.78)
(vii) Boston keratoprosthesis	3 (0.78)
(viii) Excision of ocular surface squamous neoplasia	2 (0.52)
Diabetes mellitus	45 (24.86)

**Table 2:** Probability of achieving complete corneal epithelialization during followup.

Time (day)	Probability of the events (%)	SE	95% CI
7	80.68	0.04	71.86–88.14
14	93.18	0.03	86.66–97.21
21	94.55	0.02	88.27–98.07
28	96.36	0.02	90.08–99.14
35	—	—	—
42	—	—	—
49	—	—	—
56	—	—	—
61	—	—	—

SE: standard error; CI: confidence interval.

**Table 3:** Summary of 11 patients unresponsive to serum eye drops treatment.

Diagnosis	Indication	Number (%)	Increased dosage	Further treatment
Alkali injuries	Tectonic PK	1 (0.55)	100% ASE q 1 hr	AMT with tarsorrhaphy
Ocular cicatricial pemphigoid	Therapeutic PK	1 (0.55)	100% AlloSE q 1 hr	AMT with tarsorrhaphy
Severe fungal keratitis	Therapeutic PK	2 (1.10)	100% ASE q 1 hr	AMT (2), Gunderson conjunctival flap (1)
Stevens-Johnson syndrome				
(i) Corneal ulcer failed medical therapy	Therapeutic PK	2 (1.10)	100% ASE q 1 hr	AMT with tarsorrhaphy (2)
(ii) Corneal melting and perforation	Therapeutic PK	2 (1.10)	100% ASE q 1 hr	AMT with tarsorrhaphy (1), evisceration (1)
(iii) Post-Boston keratoprosthesis surgery	Optical	3 (1.66)	100% ASE q 1 hr	Regraft, glue adhesion, and tarsorrhaphy (2), evisceration (1)

ASE: autologous serum eye drops; AlloSE: allogeneic serum eye drops; PK: penetrating keratoplasty; AMT: amniotic membrane transplantation.
